# Associations between Diet and Changes in Pain Levels among Young Women with Premenstrual Syndrome—A Preliminary Study during the COVID-19 Pandemic

**DOI:** 10.3390/jcm12124015

**Published:** 2023-06-13

**Authors:** Małgorzata Mizgier, Grażyna Jarząbek-Bielecka, Michalina Drejza, Dawid Luwański, Małgorzata Wójcik, Katarzyna Plagens-Rotman, Tomasz Gozdziewicz, Magdalena Pisarska-Krawczyk, Witold Kędzia

**Affiliations:** 1 Department of Sports Dietetics, Chair of Dietetics, Faculty of Health Sciences, Poznan University of Physical Education, 61-871 Poznan, Poland; 2 Division of Gynaecology, Department of Gynaecology, Poznan University of Medical Sciences, 61-758 Poznan, Poland; grajarz@o2.pl (G.J.-B.);; 3 Center for Pediatric, Adolescent Gynecology and Sexology, Division of Gynecology, Department of Gynecology, Poznan University of Medical Sciences, 61-758 Poznan, Poland; plagens.rotman@gmail.com; 4 Department of Obstetrics and Gynaecology, Cambridge University Hospitals NHS Foundation Trust, Cambridge CB2 0QQ, UK; 5 Department of Physiotherapy, Faculty of Sport Sciences in Gorzow Wielkopolski, Poznan University of Physical Education, 61-871 Poznan, Poland; 6 Nursing Department, President Stanislaw Wojciechowski State University of Kalisz, 62-800 Kalisz, Poland

**Keywords:** COVID-19 pandemic, premenstrual syndrome, women’s health, diet, nutrition

## Abstract

The aim of this study was to investigate the association between PMS (premenstrual syndrome)-related pain among young women following a particular type of diet during the COVID-19 pandemic. This was compared to the period before the pandemic. Furthermore, we aimed to determine whether the increase in the intensification of pain was correlated to their age, body weight, height and BMI, and whether there are differences in PMS-related pain between women who differ in their diet. A total of 181 young female Caucasian patients who met the criteria for PMS were involved in the study. Patients were divided according to the kind of diet they had followed during the last 12 months before the first medical evaluation. The rise in pain score was evaluated according to the Visual Analog Scale before and during the pandemic. Women following a non-vegetarian (“basic”) diet had a significantly higher body weight in comparison to those on a vegetarian diet. Furthermore, a significant difference was noted between the level of intensification of pain before and during the pandemic in women applying a basic diet, a vegetarian and an elimination diet. Before the pandemic, women from all groups felt weaker pain than during the pandemic. No significant difference in the intensification of pain during the pandemic was shown between women with various diets, nor was there a correlation between intensification of pain and the girl’s age, BMI, their body weight and also height for any of the diets applied.

## 1. Introduction

Premenstrual syndrome (PMS, Premenstrual Syndrome) and Premenstrual Dysphoric Disorder (PMDD) are characterized by the occurrence of cyclically repeated mental, physical and behavioral disorders that appear in the luteal phase and disappear in the follicular phase of the menstrual cycle. According to the ACOG (American College of Obstetricians and Gynecologists), PMS is defined as the occurrence of at least one somatic symptom and one mental symptom [1]. Whereas, according to the American Psychiatric Association’s DSM-5 (Diagnostic and Statistical Manual of Mental Disorders, 5th ed.) PMDD is defined as occurring whenfive symptoms present, including at least one mental symptom [2]. The typical mental symptoms include mood swings, depressed mood, anxiety, sluggishness and a feeling of “loss of control”. Frequent physical symptoms include breast engorgement and tenderness, flatulence, indigestion, headaches with the features of migraine, reduction in the ability to perform visuospatial analysis and in cognitive abilities are among the behavioral symptoms [3,4,5].

Intensified symptoms are observed in between 2.5 and 5% of women aged 25–35. However, cyclical complaints related to the menstrual cycle can concern even 40–80% of girls and women. These symptoms usually intensify after childbirth and disappear after menopause [6,7].

Although many studies have been conducted to date, the etiology of PMS is yet to be sufficiently explained [4,8,9]. However, the evidence is clear that the symptoms of PMS have a negative influence on the quality of life of the women burdened. PMS symptoms are restricting women’s daily activity related to school and work [10,11].

Scientific sources state that the pathogenesis of PMS is related to the hormonal cycle of the ovaries and changes in the concentration of some hormones (a low concentration of progesterone and higher concentrations of estradiol) to which women with PMS are more “sensitive” than women in whom PMS has not been confirmed.

Another theory discusses the role of neurotransmitters such as serotonin and gamma-aminobutyric acid (GABA) [12,13]. Serotonin receptors react to estrogen and progesterone, therefore, selective serotonin reuptake inhibitors (SSRI) have the capacity to reduce the symptoms of PMS. The level of GABA is modulated by allopregnanolone, which is a metabolite of progesterone. It has been established that the concentration of this metabolite in female patients with PMS seems to be decreased [14,15,16].

The development of PMS can be associated with the occurrence of stressful life events. It is proven that PMS is related to the incorrect influence of nerve mediators on the steroid activity of the ovaries in the central nervous system. As already mentioned, serotonin and GABA are most likely to be the greatest contributors. GABA receptors are responsible for such aspects as control of fear with the participation of the neuroactive metabolite progesterone. Modulators of receptors, in the case of the low concentrations that occur in PMS, cause intensified fear and anxiety (anxiogenic properties). The results of research on serotonin indicate the existence of a connection between the serotonin concentration and changes in mood and appetite and the modulation of serotonin activity using steroid sex hormones. In women suffering from PMS, in comparison to the control group, a significantly lower concentration of serotonin in the blood is confirmed, which can stem from an impaired postsynaptic serotonergic reaction [17,18,19].

The COVID-19 pandemic and related restrictions can be counted among the factors that intensified mental stress and, therefore, the increase in premenstrual symptoms [20]. Furthermore, it is proven that the COVID-19 pandemic, the accompanying lockdowns, subsequent lack of physical activity, the occurrence of negative emotions and deterioration of mental health, all are associated with the growth in BMI (Body Mass Index) and the occurrence of obesity. At the same time, these factors intensify mental stress, thus, generating a vicious circle [21].

The diet-related factors that can influence PMS symptoms and impact on serotonin concentration include an excessive intake of carbohydrates and low intake of protein, which causes an increase in the serotonin concentration and its release in the brain. This in turn leads to significant consumption of tryptophan, which is its precursor. Additionally, deficiencies in vitamins from the B group (B1, B2 and B6), vitamin D and magnesium, as well as changes in the metabolism of glucose, can have a significant impact on PMS [6]. It is also proven that intensified symptoms of PMS can be related to excessive consumption of tea, coffee, cola-type beverages and alcoholic beverages, in addition to chocolate, food abounding in monosaccharides, excess sodium [22] and calcium deficiency [23].

With regard to this, the aim of our research was to check if the intensification of PMS-related pain observed in young women who differed in their diet during the COVID-19 pandemic is different in comparison to the period before the pandemic. Another aim was to ascertain whether the increase in the intensification of PMS-related pain in young women is correlated with the age and anthropometric parameters of these patients and whether there are differences in the intensification of pain between women following various kinds of diets.

We hypothesized that the intensification of PMS-related pain was higher at the time of the pandemic, rather than before, in women following various kinds of diets, and that the intensification of pain during the pandemic differs in women representing various kinds of diets and is dependent on body weight, growth and BMI.

## 2. Materials and Methods

### 2.1. Participants

This case-control study was conducted twice, i.e., before the COVID-19 pandemic and during the pandemic, from 2019 to 2021 among female patients at the Division of Adolescent Gynecology and Sexology in the Department of Perinatology and Gynecology at Poznan University of Medical Sciences, who reported for examinations due to pain related to PMS. A total of 181 patients who fulfilled criteria of PMS were involved in the study. All patients were Caucasian, median age was 17.5. They were COVID-19-free on the day of hospital visit, which was confirmed with the use of COVID-19 antigen tests. The PMS diagnosis was based on medical evaluation by the gynecologist, checking against the diagnosis criteria (G.J.-B). Purposive sampling technique was used for sample selection.

The occurrence of other diseases that can present with similar symptoms to PMS, i.e., depression, dysthymia and generalized anxiety disorder, personality disorders, mental disorders, thyroid disorders, endometriosis, diseases due to self-aggression and allergy were among the exclusion criteria. Furthermore, any reports during the interview of irregular menstruations, taking hormonal medicines such as oral contraceptives, medicines that can have an impact on the functioning of the axis hypothalamus—pituitary—gonads (HPG) axis and dietary supplements were also taken into account [3,4,17,24].

### 2.2. Medical Evaluation

The premenstrual syndrome (PMS) was diagnosed on the basis of American College of Obstetricians and Gynecologists’ (ACOG) diagnostic criteria [1]. PMS was diagnosed when at least one of the listed somatic or mental symptoms had occurred in the luteal phase (in the period from 5 days before occurrence of bleeding) to the 4th day of the follicular phase (i.e., the 4th day of menstruation). Symptoms were evaluated on the basis of a dual Daily Record of Severity of Problems (DRSP) kept by female patients (before and during the COVID-19 pandemic). Prior to this study, the questionnaire was translated into Polish by two independent translators. The possible translation discrepancies were eliminated after consultations and vetting with the study team. It was also piloted with 20 patients prior to the study.

The participants were asked to keep a diary of daily activities for two consecutive menstrual cycles. During these observations, each participant was asked to rate their score of PMS-related symptoms and its impact on their social functioning [1,2,4,25].

A range from 1 to 6 was used to evaluate the level of symptoms related to PMS, where 1 meant—not at all (lack of pain), 2—minimal, 3—mild, 4—moderate, 5—heavy and 6—extreme pain.

In the columns for the subsequent days of menstruation, the patient entered an evaluation in the range 1–6 and the sum of evaluations related to the intensification of symptoms connected with PMS. PMS was diagnosed, if:

- the sum of evaluations was higher than 50 during two consecutive menstrual cycles.
- more than three items had an average result over 3 (mild) in the luteal phase, adding results of five-day intervals during the luteal and follicular phase.
- the result of the luteal phase was 30% higher than in the follicular phase [6,7,14,26].

During extended interview and medical evaluation, all the women examined declared that before the pandemic, and during the pandemic none of them did any exercise, nor did they attend acupuncture, acupressure or reflexology treatments. All the women declared having a sedentary lifestyle.

### 2.3. Dietary Assessment

Patients were qualified by diet followed during the last 12 months before pandemic. They were grouped according to diet as follows: basic (*n* = 143), elimination (*n* = 22) or vegetarian (*n* = 16). The patient’s diet was assessed based on the questions regarding the eliminated products the patients were asked about during the medical evaluation before pandemic. At the second medical evaluation, during the pandemic, the patients declared that they did not change their diet and nutrition habits.

The group with a basic diet included girls and women who did not eliminate any nutrients from their diet. The elimination diet group comprised patients who eliminated food products containing gluten, i.e., products that contained wheat, rye, barley and oat or/and milk and dairy products containing lactose. The vegetarian diet group included women that had eliminated meat and meat products or, additionally, one or some from the following products: fish, eggs and milk products.

### 2.4. Anthropometric Assessment

All women taking part in the study were measured and weighed to the nearest 1 mm and 0.2 kg, respectively, using SECA 899 medical scales and a SECA 217 stadiometer. BMI (Body mass index) was calculated as the ratio of body weight in kilograms to height squared, expressed in meters (kg/m^2^).

### 2.5. Evaluation of Pain Score

This study used VAS (Visual Analog Scale), which is designed for evaluating pain that is experienced, in which 0 means a total lack of pain and 10 means unbearable pain (the strongest pain that the patient can imagine). VAS is a validated tool for the quick and easy evaluation of the intensification of symptoms of PMS-related pain in medical practice [27,28,29]. The patients noted down the intensification of pain twice (before and during the pandemic), using the same analogue VAS for evaluating the intensification of pain.

Cyclically repeated measurements of pain intensification using the VAS enable the evaluation of changes in the pain intensification scale. Increased intensification of pain on the VAS (∆ VAS) was determined as the difference between the intensification of pain during and before the pandemic.

In order to obtain reliable results, during the medical visit the physician conducting the examinations ensured that the patients understood in what way they should use the scale.

### 2.6. Ethics and Dissemination

Before the examinations, all patients were informed about the course, procedures and aims of these examinations, and signed a consent form to participate in the study. Examinations were conducted according to the Declaration of Helsinki and accepted by the Bioethics Committee at the Medical University in Poznan. They were registered under the ID number: 553/18 of 14 June 2018 with appendix number 1 of 13 February 2020.

### 2.7. Statistical Analyses

Statistical calculations were performed using STATISTICA 10 PL statistical software. Measurable variables (body weight, height, BMI and intensification of pain on the VAS) were described using basic parameters: arithmetic mean, standard deviation (SD), and median, minimal and maximal value (min. and max.).

Due to the non-normal distribution of measurable variables, non-parametric tests were used for statistical analysis:

- Kruskal–Wallis test—to check the significance of the difference in three groups of diets.
- Wilcoxon signed rank test—to check the significance of the difference before and during the pandemic (dependent trials)
- Spearman’s rank correlation coefficient test—to study the correlation between various measurable variables.

*p* < 0.05 was acknowledged as statistically significant.

### 2.8. Patient and Public Involvement

Patients and/or the public were not involved in the design, or conduct, or reporting or dissemination plans of this research.

## 3. Results

A total of 181 young women suffering from PMS took part in this study. The patients were divided according to the kind of diet they had followed during the last 12 months. A total of 79% of them (143 women) declared to have followed a basic diet, 8.8% (16 women) a vegetarian diet and 12.2% an elimination diet.

The Kruskal–Wallis test did not reveal any significant difference in age between the basic, vegetarian and elimination diet group of patients, in height between patients in the three groups as well as in BMI [Table 1]. However, we have observed a significant difference in body weight between the groups examined (*p* = 0.0421) [Table 1]. A test of repeated comparisons (post hoc type) showed that the women on the basic diet had a significantly higher body weight in comparison with those on a vegetarian diet (*p* = 0.0413) [Table 2].

The Kruskal–Wallis test did not reveal a significant difference between women with various diets in terms of the level of intensification of pain on the VAS either before the pandemic or during it (*p* > 0.05) [Table 3]. The Wilcoxon signed rank test showed a significant difference in the pain intensification level before and during the pandemic in women on a basic (*p* < 0.0001), vegetarian (*p* = 0.0015) and elimination (*p* = 0.0003) diet. Before the pandemic, women from all groups experienced weaker pain than during the pandemic [Table 4].

### Increase in the Intensification of Pain on the VAS (∆ VAS)

The Kruskal–Wallis test did not show a difference in the increase in the intensification of pain on VAS *(*∆ *VAS)* among women with various diets (*p* > 0.05) [Table 5].

Furthermore, Spearman’s rank correlation coefficient did not show a significant correlation between the women’s BMI and ∆ VAS for the three diets—basic, vegetarian and elimination. Similarly, no significant correlation was shown between the patients’ age, body weight and height and ∆ VAS for any of the diets applied (*p* > 0.05) [Table 6].

## 4. Discussion

In the last few years, in gynecological practice there has been an increase in pain levels related to premenstrual syndrome and menstruation among patients. However, given that the scientific data indicate that PMS can also relate to nutrition [6,22,23], body weight and BMI [4], the authors of this study decided to check whether anthropometric parameters of female patients can correlate with the intensification of PMS-related pain and if the increase in the intensification of pain during the pandemic is different between women who differ in their diet.

The symptoms of PMS often lead to dysmenorrhea that lasts during the first 1–2 days of the cycle. The pain is located in the abdomen and spreads to the sacral region of the backbone and the groin area. In order to distinguish pain related to painful ovulation, which can be a symptom of primary or secondary painful menstruation, from pain that can result from PMS, a Daily Record of Severity of Problems (DRSP) [4] recommended by the ACOG was used in our study to diagnose PMS [27,29].

Our research indicates that during the pandemic the women who were examined, independent of the diet followed (basic, elimination or vegetarian), experienced stronger PMS-related pain than before the pandemic. This is probably related to psychosomatic reactions connected with the stress brought about by the COVID-19 pandemic. It is worth mentioning that chronic stress decreases immunity and thus increases susceptibility to infection, including SARS-CoV-2. An additional factor that can have a negative impact on immunity is lack of physical activity, which was related to temporary limitations on leaving home during the pandemic (numerous “lockdowns”). Regular, moderate physical activity reduces susceptibility to stress and various diseases. Physical activity in the form of swimming [30], Pilates [31], aerobics or yoga [32] can also have a significant impact on alleviating PMS-related pain. A study by Vaghela et al. looked at the influence of aerobic exercise and yoga on the intensification of PMS-related pain on the VAS. For this purpose, 72 women with an average age of 28 years were recruited and divided into two groups. One group was assigned to yoga exercises and the other to aerobic exercises. In both groups, the intensification of pain was measured on the VAS before the research started, then after two and four weeks of the study. The research findings indicated that both aerobic exercises and yoga significantly reduced the intensity of pain and the symptoms of PMS. However, yoga proved to be more effective than aerobic exercises in alleviating the symptoms of PMS. This was probably due to its strong impact on soothing stress reaction and the focus on relaxation emphasized during certain yoga classes. This is particularly evident if yoga is used in conjunction with acupressure or/and acupuncture. These treatments, such as reflexology interventions on the feet, applied independently of exercises, are equally effective in reducing the mental and physical symptoms of PMS [33,34,35]. In our study, all participants were asked about their level of physical activity. Interestingly, all the women declared that before the pandemic and during the pandemic none of them performed any exercise, nor did they attend acupuncture, acupressure or reflexology treatments. All the women declared leading a sedentary lifestyle.

Psychosocial stress is a risk factor for the symptoms of premenstrual tension and as previous research indicates, women with PMS are more susceptible to experiencing stress than those without this syndrome [12,13]. A study by Beddig et al. showed that women in whom symptoms of premenstrual tension are established, as opposed to the healthy control group, were more exposed to the negative impact of daily stress factors, which in turn influenced the intensification of mental health symptoms in the late luteal phase of their cycle in relation to the follicular phase [36].

Stress can be caused by mental, physical and sexual factors, including those related to maltreatment in childhood, which has been proven to influence the occurrence of depression in later life. In a study by Younes et al., the association between stress, mental and sexual maltreatment in childhood was analyzed. The study found that intensified symptoms of premenstrual dysphoric disorder (PMDD), depression caused by earlier traumatic experiences, were found as an intermediary factor [37]. As many authors indicate, stress related to the COVID-19 pandemic may be a factor causing depression and thus influencing overall health and well-being, including gynecological health [38,39,40]. The aim of a study conducted by Takeda et al. was to show a connection between the symptoms of premenstrual tension and stress caused by the COVID-19 pandemic. A total of 871 young, regularly menstruating women took part in this study. Study participants were asked to complete a Premenstrual Symptoms Questionnaire (PSQ), and the level of stress related to the pandemic was also measured. Symptoms related to PMS were significantly stronger in women with a higher level of stress connected with COVID-19 [20]. At the time of the pandemic, there were more frequent problems related to psychosomatic and sexual dysfunctions in women. Moreover, more symptoms of depression and anxiety were observed, which can intensify problems related to premenstrual tension and menstruation. The need to remain at home during the pandemic for a prolonged time has significantly increased feelings of loneliness amongst women. Furthermore, many women experienced an additional burden related to childcare and the need to supervise children during remote learning while simultaneously performing their own professional work or continuing studies when socially distancing. Additionally, there is evidence that domestic violence was on the rise during the pandemic, which might have also contributed to increased stress levels. Therefore, it is undisputed that the COVID-19 pandemic influenced women’s health, including the experience of PMS-related pain [38].

During the pandemic, a need for proper nutrition and a balanced diet were emphasized as a way to maintain a strong immune system. A balanced diet in combination with other factors of a healthy lifestyle, including physical activity and low level of stress, can contribute to fighting off infections. It also helps to maintain the appropriate body weight and BMI. In addition, it has an indirect influence on the appropriate course of menstruations without pain [41,42]. Following a diet with a high proportion of meat and meat products, and simultaneously deficient in vegetables and fruits and wholemeal grain products, has a negative impact on the composition of the intestinal flora, causing inflammation, oxidative stress and an overall decrease in immunity [43]. It was observed that a low plant-based protein intake and a high intake of animal-based proteins is related to the more frequent occurrence of PMS. In contrast, a diet rich in vegetables and fruits, wholemeal grain products, probiotics and prebiotics is more favorable for health, including gynecological health [44,45,46] These criteria are fulfilled by a vegetarian diet based completely or partially on vegetable components, i.e., mainly vegetables, fruits rich in cellulose, nuts and leguminous plants containing plant-based protein.

A vegetarian diet which is entirely eliminating products of animal origin is a vegan diet, while other vegetarian diets are those including eggs (ovo-vegetarian diet), milk (lacto-vegetarian) or both components simultaneously (lacto-ovo-vegetarian). Consumption of vegetable diets is safe and effective in all stages of life, including pregnancy and lactation, as well as among children and adolescents [47]. Vegetarian diets that are rich in cellulose and polyphenols have a more favorable influence on the modulation of intestinal microflora, producing metabolites that have anti-inflammatory effects. Such diets also impact positively on the immune system and prevent chronic diseases such as obesity [48,49,50]. Obesity, which is a result of overnutrition and a lack of physical activity, is related to chronic inflammation and can contribute to a worse course of infection, including COVID-19, and can negatively influence sexual health [51,52].

Most studies based on a population of women with PMS aged from 18 to 44 years prove that this syndrome is observed more frequently in women with excessive body weight, i.e., BMI > 30 [53,54] and BMI > 27.5 kg [4]. At the same time, the research conducted within the scope of our study indicates a significant difference in body weight between the various groups examined. Women following a basic diet had a significantly higher body weight in comparison to women who followed a vegetarian diet (*p* = 0.0413). Moreover, a vegetarian diet not only favors maintaining a body weight within the appropriate range, but also supports the functioning of the immune system and guarantees lower morbidity from civilization diseases [47,48,50].

Nonetheless, our earlier research indicates that PMS in the group of 18-year-old patients can occur more frequently in patients with a normal body weight (BMI < 25), lower content of adipose tissue (Fat Mass), higher content of lean tissue (Fat Free Mass) and water (TBW, Total Body Water) in comparison to girls with excessive body weight. Thus, there seems to be an association between the proportion of adipose tissue, lean mass and water and PMS symptoms [6]. That is why further research should include body composition analysis. Additionally, the groups should be disaggregated into different age categories.

Siminiuc and Turcanu (2023) showed that diet may be an important factor that reduces the symptoms of PMS in female patients. Unfortunately, there is very little research taking into consideration the influence of diet on PMS. No correlations were identified between PMS and the consumption of macronutrients such as protein, fat and carbohydrates high in fiber. Supplementation of calcium, magnesium, vitamin D, B vitamins and herbs might be helpful for symptomatic relief. However, the evidence is insufficient to recommend special diet composition or supplementation which may be effective and helpful in PMS treatment [55].

### Strengths and Limitations

In this study, data concerning the amount of daily calorie intake, and the macronutrient composition of the diet (before and after the pandemic) were not collected. We did not use a Food Frequency Questionnaire (FFQ) or a daily food record and body composition analysis, which might have been helpful in supporting the current evidence base. Unfortunately, the data about marital status and socio–economic status were also not collected. Furthermore, the study includes only young women. However, the strength of the study is that we examined quite a large group of young female patients who differed in their types of diet.

## 5. Conclusions

There was no difference between women with various diets in terms of the level of pain either before the pandemic or during pandemic. However, we observed that before the pandemic, women from all groups experienced significantly weaker pain than during the pandemic.

Moreover, there was no difference with regards to the level of pain on VAS (∆ VAS) among women with various diets. Furthermore, there was a correlation between the women’s age, body weight, body height, BMI and ∆ VAS either for basic and vegetarian, or for elimination diets. It should be emphasized, however, that the presented results of the study are only preliminary. In future studies on this topic, additional clinical trials are needed which would provide more rigorous scientific results.

Additionally, our research findings suggest an area for future scientific studies, i.e., searching for other factors that influence the intensification of PMS-related pain (including checking whether the women’s body composition, i.e., content of adipose tissue, lean tissue and water plays a role) in order to create recommendations that might prove to be of importance in the work of family doctors, gynecologists, sexologists and mental health advisers, and dieticians who encounter the problem of PMS in their practice.

## Figures and Tables

**Table 1:** Descriptive statistics of age, height, BMI and body weight in women with various diets, and results of the Kruskal–Wallis test.

Diet	*n*	Age (Years)					H	*p*
		Average	SD	Mediana	Min.	Max.		
Basic	143	20.1	7.1	17	17	21	2.59	0.2734
Vegetarian	16	20.4	5.6	17.5	16	22		
Elimination	22	21.4	7.4	20	17	22		
Total	181	20.3	7	17.5	17	21		
Diet	*n*	Height (cm)					H	*p*
		Average	SD	Median	Min.	Max.		
Basic	143	166.4	6.5	168	147	183		
Vegetarian	16	165.7	6.8	168.5	154	175	0.1	0.9494
Elimination	22	167.1	7.3	167	155	179		
Total	181	166.4	6.6	168	147	183		
Diet	*n*	BMI					H	*p*
		Average	SD	Median	Min.	Max.		
Basic	143	23.61	5.94	22.19	14.87	50.2		
Vegetarian	16	20.27	4.88	19.6	13.49	33.61	5.88	0.0528
Elimination	22	24.08	6.7	21.35	15.57	40.7		
Total	181	23.38	6	21.8	13.49	50.2		
Diet	*n*	Body weight (kg)					H	*p*
		Average	SD	Median	Min.	Max.		
Basic	143	65.4	16.9	63	40	140		
Vegetarian	16	55.7	13.9	54	32	96	6.34	0.0421 *
Elimination	22	67.2	18.7	61.5	44	119		
Total	181	64.8	17	62	32	140		

H—value of Kruskal–Wallis test; *p*—level of probability; SD—standard deviation; Min—minimal value; Max—maximal value; *n*—number of women and * statistical significance *p* < 0.05 was marked with an asterisk.

**Table 2:** The differences in body weight between patients on basic vs. vegetarian, basic vs. elimination and vegetarian vs. elimination diets.

	Basic vs. Vegetarian	Basic vs. Elimination	Vegetarian vs. Elimination
Level *p*	0.0413 *	1.000	0.0986

*p*—level of probability; * statistical significance *p* < 0.05 was marked with an asterisk.

**Table 3:** Descriptive statistics for the intensification of PMS-related pain on the VAS before and during the pandemic in women with various diets, and the results of the Kruskal–Wallis test.

Before the Pandemic								
Diet	N	VAS					H	*p*
		Average	SD	Median	Min.	Max		
Basic	143	5.7	1.7	6.0	3	10	1.23	0.5398
Vegetarian	16	6.3	1.8	6.0	4	10		
Elimination	22	5.8	1.7	5.5	3	8		
During the Pandemic								
Diet	N	VAS					H	*p*
		Average	SD	Median	Min.	Max.		
Basic	143	5.7	1.7	6.0	3	10	1.23	0.5398
Vegetarian	16	6.3	1.8	6.0	4	10		
Elimination	22	5.8	1.7	5.5	3	8		

PMS-premenstrual syndrome; VAS—visual analog scale; H—value on the Kruskal–Wallis test, *p*—level of probability; SD—standard deviation; Min—minimal value Max.—maximal value; *n*—number of women.

**Table 4:** Descriptive statistics of the intensification of PMS-related pain on the VAS before and during the pandemic in groups of women with various diets, and the results of the Wilcoxon signed rank test.

Basic Diet								
Period	*n*	VAS					Z	*p*
		Average	SD	Median	Min.	Max.		
Before pandemic	143	5.7	1.7	6.0	3	10	9.47	<0.0001 *
During pandemic	143	7.3	1.5	8.0	4	10		
Vegetarian Diet								
Period	*n*	VAS					Z	*p*
		Average	SD	Median	Min.	Max.		
Before pandemic	16	6.3	1.8	6.0	4	10	3.18	0.0015 *
During pandemic	16	8.0	1.4	8.0	5	10		
Elimination diet								
Period	*n*	VAS					Z	*p*
		Average	SD	Median	Min.	Max.		
Before pandemic	22	5.8	1.7	5.5	3	8	3.62	0.0003 *
During pandemic	22	7.4	1.5	8.0	4	10		

PMS-premenstrual syndrome; VAS—visual analog scale; * Statistical significance *p* < 0.05 was marked with an asterisk; *n*—number of women; Z—value of Wilcoxon signed rank test and *p*—level of probability.

**Table 5:** Descriptive statistics of the increase in the intensification of PMS-related pain on VAS in women with various diets, and the results of the Kruskal–Wallis test.

Diet	*n*	Δ VAS					H.	*p*
		Average	SD	Median	Min.	Max.		
Basic	143	1.6	1.2	1.0	0	5	0.15	0.9268
Vegetarian	16	1.8	1.3	2.0	0	4		
Elimination	22	1.6	1.3	2.0	0	4		

(∆ VAS)—increase in the intensification of pain on VAS (Visual Analog Scale); H—value of the Kruskal–Wallis test; *p*—level of probability; SD—standard deviation; Min—minimal value; Max—maximal value; *n*—number of women.

**Table 6:** Results of Spearman’s rank correlation coefficient test of significance between age, BMI, and body weight and height in women and the increase in the intensification of PMS-related pain on the VAS (∆ VAS).

Pair of Variables	Diet	*n*	Rs	*p*
Δ VAS & Age (years)	Basic	143	−0.064	0.4472
	Vegetarian	16	−0.042	0.8761
	Elimination	22	−0.142	0.5273
	Basic	143	−0.062	0.4588
∆ VAS & BMI (kg/m^2^)	Vegetarian	16	0.269	0.3138
	Elimination	22	−0.028	0.8998
	Basic	143	−0.078	0.3559
∆ VAS & Body weight (kg)	Vegetarian	16	0.339	0.1993
	Elimination	22	−0.033	0.8855
	Basic	143	−0.116	0.1677
∆ VAS & Height (cm)	Vegetarian	16	−0.165	0.5420
	Elimination	22	−0.058	0.7993

(∆ VAS)—increase in the intensification of pain on the VAS (Visual Analog Scale); Rs—value of Spearman’s rank correlation coefficient; *p*—level of probability and *n*—number of women.

## Data Availability

Data were obtained without consent for sharing and therefore may not be made available. No data are available.
